# Examining impairment and kinetic patterns associated with recent use of hemp-derived Δ^8^-tetrahydrocannabinol: case studies

**DOI:** 10.1186/s42238-022-00146-9

**Published:** 2022-07-07

**Authors:** Gregory T. Wurz, Edward Montoya, Michael W. DeGregorio

**Affiliations:** 1 RCU Labs, Inc., 408 Sunrise Avenue, Roseville, CA 95661-4123 USA; 2 Cancer Immunotherapy Research Institute, 408 Sunrise Avenue, Roseville, CA 95661-4123 USA

**Keywords:** Δ^8^-THC, ∆^9^-THC, Impairment, Nystagmus, Hemp

## Abstract

**Background:**

As a result of the legalization of U.S. industrial hemp production in late 2018, products containing hemp-derived Δ^8^-tetrahydrocannabinol (Δ^8^-THC) are increasing in popularity. Little, however, is known regarding Δ^8^-THC’s impairment potential and the associated impacts on roadway and workplace safety, and testing for Δ^8^-THC is not yet common. The present study explored impairment patterns and cannabinoid kinetics associated with recent use of Δ^8^-THC.

**Methods:**

Hemp-derived Δ^8^-THC concentrate was administered by vaporization ad libitum to three male frequent cannabis users aged 23–25 years. In addition to self-assessments of impairment using a 10-point scale, horizontal gaze nystagmus (HGN) was evaluated in each subject as a physical means of assessing impairment before and after vaporization. To examine cannabinoid kinetic patterns, exhaled breath and capillary blood samples were collected prior to vaporization up to 180 min post-vaporization and analyzed by liquid chromatography high-resolution mass spectrometry for cannabinoid content using validated methods. The impairment and cannabinoid kinetic results were then compared to analogous results obtained from the same three subjects after they had smoked a ∆^9^-THC cannabis cigarette ad libitum in a previous study to determine whether any similarities existed.

**Results:**

Patterns of impairment after vaporizing Δ^8^-THC were similar to those observed after smoking cannabis, with self-assessed impairment peaking within the first hour after use, and then declining to zero by 3 h post-use. Likewise, HGN was observed only after vaporizing, and by 3 h post-vaporization, evidence of HGN had dissipated. Cannabinoid kinetic patterns observed after vaporizing Δ^8^-THC (short ∆^8^-THC half-lives of 5.2 to 11.2 min at 20 min post-vaporization, presence of key cannabinoids cannabichromene, cannabigerol, and tetrahydrocannabivarin, and breath/blood Δ^8^-THC ratios > 2 within the first hour post-vaporization) were also analogous to those observed for ∆^9^-THC and the same key cannabinoids within the first hour after the same subjects had smoked cannabis in the previous study.

**Conclusions:**

Hemp-derived Δ^8^-THC and Δ^9^-THC from cannabis display similar impairment profiles, suggesting that recent use of Δ^8^-THC products may carry the same risks as cannabis products. Standard testing methods need to incorporate this emerging, hemp-derived cannabinoid.

## Introduction

**Fig. 1:**
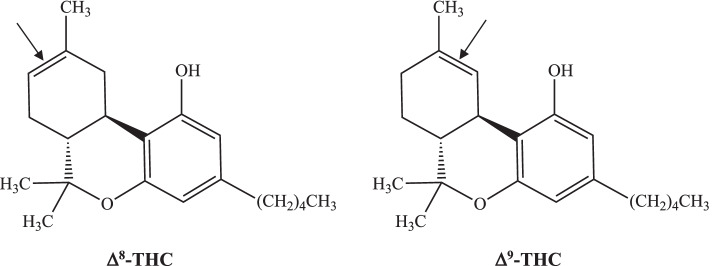
Chemical structures of Δ^8^-THC and Δ^9^-THC. Arrows point out the position of the double-bond in the cyclohexene ring of each molecule

Δ^8^-Tetrahydrocannabinol (Δ^8^-THC) is a positional isomer of the much more common Δ^9^-THC, which is the main psychoactive component of the cannabis plant (*Cannabis sativa*), differing only in the location of a carbon-carbon double bond (see Fig. 1). Compared to Δ^9^-THC, the Δ^8^-THC isomer is far less abundant, representing less than 1% of total THC, and like cannabinol (CBN) it occurs as a degradation product of Δ^9^-THC (Hazekamp et al. 2010; Hazekamp et al. 2016), with no evidence to support natural synthesis of Δ^8^-THC by the plant. Given its low natural abundance in plant material, large quantities of ∆^8^-THC are being chemically synthesized from hemp-derived cannabidiol (CBD), a process for which was originally described by Mechoulam and colleagues in 1966 (Gaoni and Mechoulam 1966) and later improved and patented (Webster et al. n.d.). ∆^8^-THC has been reported to be less psychoactive compared to ∆^9^-THC (Razdan 1986; Hollister and Gillespie 1973); however, Huffman et al. found these two molecules to be nearly equipotent at the CB_1_ and CB_2_ cannabinoid receptors (Huffman et al. 1999; Bow and Rimoldi 2016), while Radwan et al. reported ∆^9^-THC to have a CB_1_ receptor affinity four times that of ∆^8^-THC, which had an affinity for the CB_2_ receptor 3.5 times that of ∆^9^-THC (Radwan et al. 2015). Clearly, more research is needed. Regardless of their relative potency, ∆^8^-THC and ∆^9^-THC are pharmacologically very similar, with ∆^8^-THC still producing a high in users and possessing many of the medicinal qualities (Hazekamp et al. 2010; Abrahamov et al. 1995; Kruger and Kruger 2021; Kruger and Kruger 2022) of ∆^9^-THC with perhaps fewer adverse effects (Kruger and Kruger 2021; Kruger and Kruger 2022).

Following federal legalization of industrial hemp in the USA in late 2018, products containing Δ^8^-THC synthesized from hemp-derived CBD have become very popular. Due to the relative lack of regulation compared to traditional cannabis products containing ∆^9^-THC, ∆^8^-THC products are widely available outside of authorized dispensaries, wherever hemp and CBD products are sold throughout the United States, including gas stations, bodegas, head shops, and online retailers. A wide variety of products containing ∆^8^-THC are available for oral consumption, e.g., gummies, tinctures, oils, chocolates, and drink mixes, for smoking, e.g., ∆^8^-THC-infused pre-rolls, and for vaporization, e.g., vape cartridges and vape pens, all of which are used in the same manner as ∆^9^-THC-containing cannabis products (Oleinik 2021; Babalonis et al. 2021). Because they are derived from hemp, these products are perceived as being legal; however, their legality is now an open question in light of the U.S. Drug Enforcement Administration’s (DEA) August 21, 2020 interim final rule, which considers all synthetically-derived tetrahydrocannabinols, which would include Δ^8^-THC, as Schedule I controlled substances regardless of their Δ^9^-THC concentration. Indeed, as of August 2021, 18 U.S. states have already either restricted or banned ∆^8^-THC, and additional states have such actions under consideration (Malyshev and Ganley 2021). Interestingly, in a ruling filed May 19, 2022, the U.S. Court of Appeals for the Ninth Circuit in California upheld ∆^8^-THC as a legal, hemp-derived product under the 2018 Farm Bill. With the increasing popularity of products containing hemp-derived Δ^8^-THC, and the relative lack of knowledge surrounding their psychoactive properties compared to products containing Δ^9^-THC, the potential roadway and workplace safety impacts of these products need to be considered, and drug testing methods need to incorporate this previously obscure cannabinoid.

Given the novelty of hemp-derived Δ^8^-THC products, there is a great deal of confusion regarding what exactly Δ^8^-THC is, what its effects are, and how it compares to Δ^9^-THC. The large number of available products, some of dubious origin, with a wide range of Δ^8^-THC potencies stated on the labels (Oleinik 2021) have only added to the confusion. A recent market report by a group based in Israel has revealed just how well-founded the legal and safety concerns are regarding these products (Oleinik 2021). Of the 38 products investigated in this report, 53% of them were found to have illegal levels of Δ^9^-THC ranging from > 0.3% to 15.2%, and the labeling on 34% of these products was unclear as to the Δ^8^-THC content. Incorrect amounts of Δ^8^-THC were stated on 68% of these products, leaving only 32% of the products with the correct amount of Δ^8^-THC stated on the label (Oleinik 2021).

Presented here are three clinical case studies conducted to examine the patterns of self-assessed and physical impairment (horizontal gaze nystagmus; HGN) following recent use of a commercially available, hemp-derived Δ^8^-THC product through inhalation (vaporization). The chosen product had a defined Δ^8^-THC content that was verified by our laboratory. These impairment patterns were then compared to those obtained in a previous cannabis smoking study in the same three subjects to determine whether impairment induced by Δ^8^-THC is similar to that induced by Δ^9^-THC. Also examined were the cannabinoid kinetic patterns in exhaled breath and blood to assess similarities with Δ^9^-THC.

## Methods and materials

### Clinical case studies

Three healthy adult subjects who had previously participated in an RCU Labs-sponsored cannabis smoking study were recruited for this hemp-derived Δ^8^-THC vaporization study. All subjects received financial compensation ($200) for their participation. This study was performed under a clinical protocol approved by the Cancer Immunotherapy Research Institute IRB (Federal Wide Assurance number FWA00029851). Written informed consent was obtained from all subjects prior to their participation, and a copy of the signed informed consent form was provided to each subject.

#### Inclusion criteria

To be included, a subject must have been a male or female cannabis user, defined as having used cannabis at least once in the past week, at least 21 years of age who had participated in a previous cannabis smoking study sponsored by RCU Labs (DeGregorio et al. 2021). Prior to their scheduled participation, they must have used cannabis within the previous 24 h, but not within the last 12 h. Upon entry, subjects were asked to complete a questionnaire requesting their age, sex, race, height, weight, cannabis use history (time since last use, number of days used in the last 14 days, how often they use cannabis, and number of years of cannabis use), their primary route of cannabis use, whether or not they use tobacco and alcohol, and any medications or supplements they are taking. Subjects were questioned regarding their medical history, and current medical problems were grounds for exclusion. Information regarding subject selection and eligibility criteria for the previous ∆^9^-THC cannabis smoking study can be found in DeGregorio et al. (DeGregorio et al. 2021).

#### Δ^8^-THC administration

After giving written informed consent, each subject was provided with a disposable vaporization device containing 1 g of hemp-derived Δ^8^-THC concentrate and instructed to vaporize the product for 5 min or until they felt maximally impaired (incapacitated), whichever occurred first, inhaling the vapor ad libitum. See “Δ^8^-THC supplies” section below for detailed information regarding the product used, and refer to “Self-assessment of impairment” section for self-assessed impairment information. Subjects were not blinded to the product being consumed (open-label study).

#### ∆^9^-THC cannabis administration

In the prior ∆^9^-THC cannabis smoking study in which the subjects had participated (DeGregorio et al. 2021), each subject was given a single cannabis cigarette and instructed to smoke as much of it as possible within a 10-min period ad libitum. The cigarettes contained 500 mg of dried cannabis flower with a ∆^9^-THC content of 24.6% by weight, and they were prepared immediately before each smoking session. To examine effects of potency on impairment, one subject (#103) was given an additional, lower potency cannabis cigarette containing 8.5% ∆^9^-THC by weight to smoke ad libitum 1 h prior to smoking the higher potency cigarette. Subjects were not blinded to the product being consumed. Cannabis supplies were legally obtained from a licensed retail establishment in the Sacramento, CA region.

#### Blood collection schedule

To establish baseline cannabinoid levels, one capillary blood sample was collected prior to vaporization. Additional blood samples were collected immediately after each subject finished vaporizing, and then at 20 min and 180 min post-vaporization, for a total of four blood samples. In the previous ∆^9^-THC cannabis smoking study, blood samples were collected from the same subjects prior to smoking and at 60 min, 180 min, and 200 min post-smoking. Samples were stored at approximately 4 °C for a maximum of 24 h before analysis. Capillary blood (100–400 μL) was collected from the upper arm using two types of automated collection devices, one purchased from Seventh Sense Biosystems (Medford, MA), and one purchased from Tasso, Inc. (Seattle, WA). The TAP I blood collection device (Blicharz et al. 2018) from Seventh Sense Biosystems is designed to collect up to approximately 130 μL over a period of 1–3 min, and the Tasso+ device manufactured by Tasso, Inc. (Hendelman et al. 2021) can collect up to approximately 400 μL in 5 min. These devices were used according to the manufacturers’ instructions. Both devices contained lithium heparin as an anticoagulant.

#### Breath collection schedule

To establish baseline cannabinoid levels, one exhaled breath sample was collected prior to vaporization. Two consecutive breath samples were collected from each subject immediately post-vaporization, followed by two consecutive breath samples at 15, 30, 45, 60, and 180 min post-vaporization, for a total of 13 exhaled breath samples. Consecutive samples at each time point were separated by approximately 2–3 min. All sample times were recorded. In the previous ∆^9^-THC cannabis smoking study in the same subjects, a pre-smoking breath sample was collected, followed by consecutive breath samples collected at 20 min and 40 min post-smoking and a single sample at 180 min post-smoking. The breath sample collection devices were designed by Sensabues AB (Stockholm, Sweden) and the Partnership for Clean Competition (Colorado Springs, CO). These self-contained, single-use devices contain an electrostatic polymer filter and are designed to collect approximately 20 L of exhaled breath (Himes et al. 2013; Hubbard et al. 2020). Devices were kept sealed in their original packaging until immediately before use to prevent contamination and used according to the manufacturer’s instructions.

#### Self-assessment of impairment

Subjects were asked to self-assess their level of impairment before ∆^8^-THC vaporization (baseline) and at each designated time point post-vaporization [immediately after (0 min), 15, 30, 45, 60, and 180 min, coinciding with each breath sampling] using a 10-point Likert-type scale, where zero denoted no impairment, 5 denoted moderate impairment, and 10 denoted maximal impairment (incapacitation) for that individual. In the previous ∆^9^-THC cannabis smoking study, the subjects were asked to assess their level of impairment at baseline and at 20, 40, 60, 180, and 200 min post-smoking, coinciding with breath sample collections, using the same 10-point scale.

#### Physical assessment of impairment: HGN

In this study, subjects were evaluated for HGN as a physical indicator of impairment prior to vaporization, immediately after vaporization, and then at 15, 30, 45, 60, and 180 min post-vaporization by an individual who had received prior training in administering this test by a law enforcement drug recognition expert training instructor. In the previous ∆^9^-THC cannabis smoking study, HGN was assessed prior to smoking and at 20, 40, 60, 180, and 200 min post-smoking. HGN refers to the involuntary movement or jerking of the eyes as they gaze to either side, and it is a component of standardized field sobriety testing (SFST) (Downey et al. 2016). Someone experiencing nystagmus is unaware of its occurrence. In this particular test, the subjects, while standing, were asked to keep their head still and follow a slowly moving horizontal object (ballpoint pen) positioned in front of their face using their eyes only. Both eyes were observed for lack of smooth pursuit, nystagmus at maximum eye deviation (45°) when held for 4 s, and the onset of nystagmus prior to a 45° deviation when held for 4 s, for a total of six clues. The presence or absence of resting nystagmus was also noted. Nystagmus was rated as “present” when subjects manifested at least 4/6 clues, “slight” when fewer than four clues were observed, “severe” if all six clues were observed combined with involuntary head movement, and “no HGN” when none of the six clues were observed.

### Δ^8^-THC supplies

Subjects were given Cake Gorilla Glue Hybrid hemp-derived Δ^8^-THC vaporization concentrate (1 g), manufactured by Cannagarden Co. (Santa Ana, CA), which was contained within a disposable, rechargeable vaporization device that was included with the product. The device model was a Kik D8 disposable vaporization pen manufactured by Shenzhen MinRuiKe Technology Co., Ltd. (Shenzhen, China). The certificate of analysis from BelCosta Labs (Long Beach, CA) (Laboratories 2020) stated that the product contained 93% Δ^8^-THC (w/w), trace amounts of CBN (< 0.5 mg/g), and no detection of Δ^9^-THC (limit of detection 0.04 mg/g). Analysis by RCU Labs confirmed the Δ^8^-THC content. This product was legally obtained from a licensed retail establishment in the Sacramento, CA, region.

### Analytical methods

#### Chemicals and reagents

The 10 cannabinoid analytes [Δ^9^-THC, Δ^8^-THC, CBN, CBD, cannabidiolic acid (CBDA), cannabichromene (CBC), cannabigerol (CBG), cannabigerolic acid (CBGA), Δ^9^-tetrahydrocannabinolic acid A (Δ^9^-THCA), and Δ^9^-tetrahydrocannabivarin (Δ^9^-THCV),] were obtained as certified reference materials (CRMs) manufactured by Cerilliant (Round Rock, TX). The Cerilliant CRMs were supplied as sealed, 1.0 mL glass ampules containing 1000 μg/mL of analyte in methanol. The internal standard (IS), deuterated Δ^9^-THC (Δ^9^-THC-D_3_), was manufactured by Cerilliant as a CRM and supplied in a sealed, 1.0 mL glass ampule containing 100 μg/mL of Δ^9^-THC-D_3_ in methanol. When not in use, concentrated stock solutions of these agents and working solutions made therefrom were stored at – 20 °C.

Acetonitrile, formic acid, methanol, and *n*-hexane were purchased from Thermo Fisher Scientific (Waltham, MA) and were of LC/MS grade. Ethyl acetate (Acros Organics) was purchased from Thermo Fisher Scientific and was of spectroscopy grade (> 99.5%). High purity water (18.2 MΩ) required for preparing the mobile phase and for sample extraction was produced using an EMD Millipore Simplicity water purification system. When not in use, these agents were stored at room temperature (20–25 °C). Nitrogen (N_2_), supplied as a cryogenic liquid in a 230L dewar at a purity of 99.998%, or as compressed nitrogen gas at a purity of 99.999% in T-type cylinders, was obtained from Praxair (Danbury, CT).

#### Analysis of cannabinoids in blood

Extraction and analysis of Δ^8^-THC, Δ^9^-THC, and other cannabinoids in blood was performed according to a validated method as previously described (DeGregorio et al. 2020). Briefly, 50 μL of each sample was mixed with 100 μL of high-purity water in a 1.5-mL microcentrifuge tube and spiked with 5.0 μL of IS solution (75 ng/mL Δ^9^-THC-D_3_). To extract, 500 μL of a solution containing 90% *n*-hexane and 10% ethyl acetate (v/v) was added to each sample, followed by vortexing for 30 s. Samples were then centrifuged at 9300 rcf for 10 min. The supernatant was transferred to a 16 mm × 125 mm borosilicate glass tube and evaporated to dryness under a gentle stream of nitrogen at 50 °C. Samples were reconstituted in 75 μL of a solution composed of 65% acetonitrile, 35% water, and 0.1% formic acid and analyzed by LC-HRMS. Supplies of whole blood needed for calibration standards were obtained from a cannabis-free donor and kept refrigerated (2–8 °C) up to 4 weeks.

The LC-HRMS system consisted of a Thermo Scientific Vanquish ultra-high-performance liquid chromatography (UHPLC) system and a Thermo Scientific Q Exactive Orbitrap mass spectrometer. All analytical data were collected and processed using TraceFinder version 4.1 software (Thermo Fisher Scientific). The mass spectrometer had the following settings: runtime 14 min; polarity positive; scan range 150–550 m/z; resolution 70,000 (full MS); automatic gain control (AGC) target 1.0 × 10^6^; maximum inject time (IT) 250 ms. The following tune settings were saved and loaded prior to analysis: sheath gas 12; auxiliary gas 6; sweep gas 1; spray voltage 3.5 kV; capillary temperature 320 °C; auxiliary gas heater 300 °C; all other settings default. Detection was performed by positive ion mode heated electrospray ionization (HESI). Analyte verification was based on the presence of molecular ions, at least two isotopic ions, and intensity ratios between the isotopic ions and the molecular ions. The UHPLC system was configured as follows: flow rate 0.300 mL/min; column temperature 37 °C; sample compartment 8 °C; injection volume 5.0 μL. The LC system was equipped with a Restek (Bellefonte, PA) Raptor ARC-18, 1.8 μm, 2.1 × 150 mm column. The mobile phase was composed of (A) water with 0.1% formic acid and (B) acetonitrile with 0.1% formic acid. Samples were eluted according to the following gradient: initial composition 75% B; increase to 100% B by 6 min; hold at 100% B for 3.5 min; decrease to 75% B by 10 min; hold at 75% B for 4 min; end run at 14 min.

#### Analysis of cannabinoids in exhaled breath

A previously validated analytical method for the quantification of the cannabinoids Δ^9^-THC, CBN, CBC, and Δ^9^-THCV in exhaled breath was used for the analysis of study samples. Additional cannabinoids analyzed included Δ^8^-THC Δ^9^-THCA, CBG, CBGA, CBD, and CBDA. For the preparation of calibration standards, sufficient quantities of the matrix (breath collection devices with electrostatic polymer filters) were obtained from SensAbues AB. Breath collection devices were kept at room temperature (20–25 °C) within their original packaging to prevent contamination.

Standard calibration solutions were prepared at 15X concentrations in methanol. The 15X standard calibration concentrations were 37.5, 75, 150, 375, 750, and 1500 ng/mL of all cannabinoids combined, based on final standard concentrations of 2.5, 5.0, 10, 25, 50, and 100 ng/mL following extraction. The IS solution (Δ^9^-THC-D_3_) was prepared in methanol at a concentration of 75 ng/mL. To prepare calibration standards for extraction, a sufficient number of appropriately labeled breath collection devices were placed on top of individual 16 mm × 125 mm borosilicate glass tubes with the mouthpieces facing up. After placement, 5 μL of the 75-ng/mL IS working solution and 5 μL of the appropriate 15X calibration standard solution were added directly onto the corresponding filter pad inside the breath collection device. After adding IS, approximately 3 min were allowed for the solutions to saturate the filter pads. After extraction, the final concentration of the IS was 5 ng/mL (75 μL final volume). Study samples were prepared by spiking with 5 μL IS solution.

To extract cannabinoids from the breath collection devices, 2 mL of methanol were aliquoted through each breath device and filter housing. After adding methanol, approximately 5 min were allowed for all of the solvent to pass through the filter pads. Next, two 2.5-mL aliquots of methanol were passed through the breath collection devices. Using a 60-mL syringe, approximately 120 cm^3^ of air was pushed through each device to force all residual methanol within the devices through the filter pads. The sample breath collection devices were then removed and the glass tubes were placed in an N-Evap Model 112 analytical nitrogen evaporator (Organomation Associates, Berlin, MA). The eluate was evaporated to dryness under a gentle stream of nitrogen, with the water bath temperature set to approximately 50 °C. Once evaporation was complete (approximately 15 min), samples were allowed to cool to room temperature (20–25 °C; approximately 2 min) and reconstituted by adding 75 μL of a solution containing 75% acetonitrile and 25% water with 0.1% formic acid. Samples were then transferred to a glass microinsert-equipped autosampler vial and placed in the autosampler compartment for analysis according to the method. TraceFinder software performed all required analyses. The chromatographic conditions for the analysis of cannabinoids in exhaled breath were the same as described above under “Analysis of cannabinoids in blood” section

## Results

### Case study subject demographics

Three subjects, designated #103-105, were recruited to examine patterns of self-assessed impairment and HGN following administration of hemp-derived Δ^8^-THC through vaporization (see Table 1 for detailed study participant information). None of the subjects reported any current medical conditions or use of medications or supplements, and all three subjects had previously participated in an RCU Labs-sponsored ∆^9^-THC cannabis smoking study in February 2020.

**Table 1:** Study participant information

Parameter	Subject 103	Subject 104	Subject 105
BMI (kg/m^2^)	31.9	25.7	32.3
Age (years)	25	25	23
Cannabis use history (years)	10	10	10
Prior cannabis use (# days/last 14 days)	14	14	14
Primary route of administration	Oral	Inhalation (vaporization)	Inhalation (smoking)
Time since last cannabis use (h)	16	24	12
Prior experience with ∆^8^-THC	None	None	None
Baseline blood ∆^9^-THC (ng/mL)	1.8	4.1	4.9
Baseline breath ∆^9^-THC (pg/filter pad)	1,298	255	< LOQ

*BMI* Body mass index, *∆*^*8*^*-THC* ∆^8^-tetrahydrocannabinol, *∆*^*9*^*-THC* ∆^9^-tetrahydrocannabinol, *LOQ* Limit of quantification (188 pg/filter pad)

### Baseline cannabinoid concentrations in blood and exhaled breath

Prior to vaporization, blood and exhaled breath samples were collected from each of the three subjects and then analyzed by LC-HRMS for cannabinoid content. As expected for frequent cannabis users, Δ^9^-THC was detected in the blood and breath of all three subjects. None of the subjects showed evidence of Δ^8^-THC in blood or exhaled breath prior to vaporization. In blood, Δ^9^-THC levels ranged from 1.8 to 4.9 ng/mL (see Table 1), with low levels (< 1 ng/mL) of other cannabinoids and Δ^9^-THC metabolites detected. This is an important finding because frequent users will often have detectable levels of multiple cannabinoids in their blood, but this does not constitute evidence of recent cannabis use within the impairment window. In breath, only Δ^9^-THC was detected in breath prior to vaporization (see Table 1).

### Self-assessed impairment and HGN

**Fig. 2:**
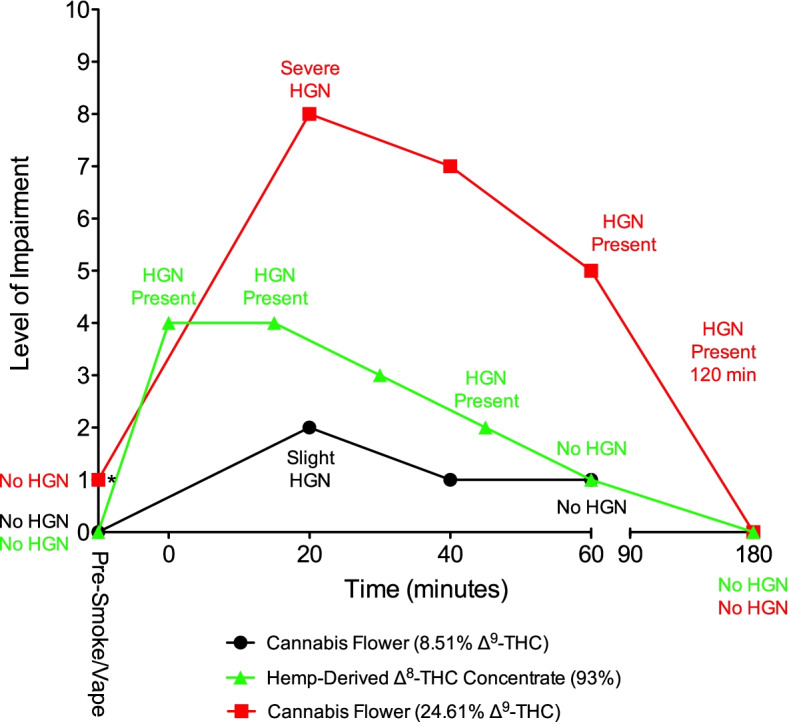
Subject 103. The levels of self-assessed impairment and the presence or absence of HGN are shown prior to and after smoking or vaporization of cannabis and hemp-derived Δ^8^-THC. *Subject smoked the high-potency cannabis flower (24.61% Δ^9^-THC) 60 min after smoking the low-potency cannabis flower (8.51% Δ^9^-THC), at which time an impairment level of “1” was reported. Evaluation time points for HGN and self-assessed impairment differed following smoking of ∆^9^-THC cannabis and vaporization of ∆^8^-THC. HGN = horizontal gaze nystagmus

**Fig. 3:**
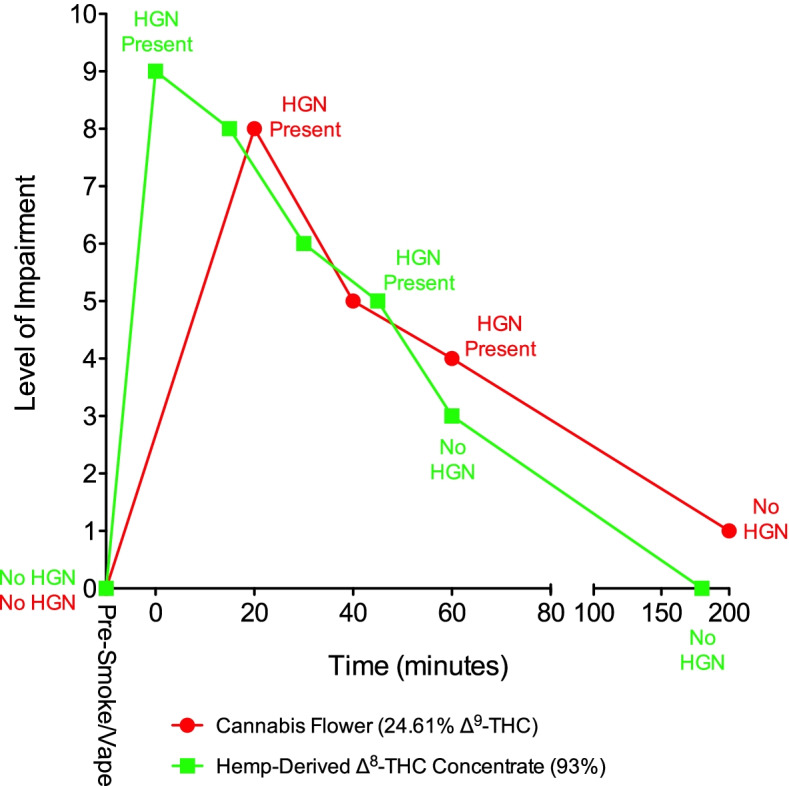
Subject 104. The levels of self-assessed impairment and the presence or absence of HGN are shown prior to and after smoking or vaporization of cannabis and hemp-derived Δ^8^-THC. Evaluation time points for HGN and self-assessed impairment differed following smoking of ∆^9^-THC cannabis and vaporization of ∆^8^-THC. HGN = horizontal gaze nystagmus

**Fig. 4:**
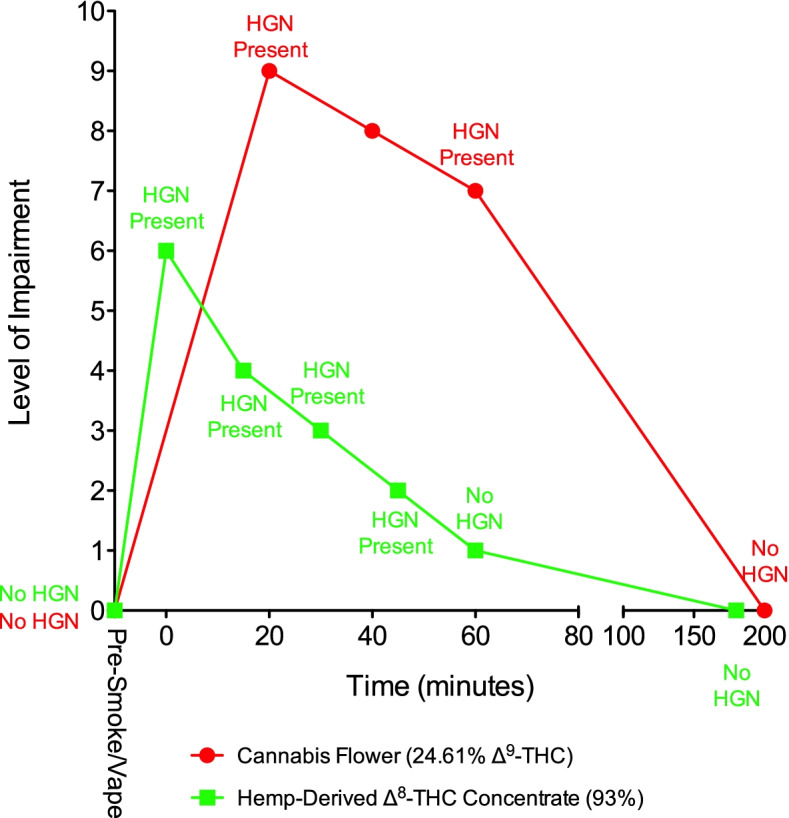
Subject 105. The levels of self-assessed impairment and the presence or absence of HGN are shown prior to and after smoking or vaporization of cannabis and hemp-derived Δ^8^-THC. Evaluation time points for HGN and self-assessed impairment differed following smoking of ∆^9^-THC cannabis and vaporization of ∆^8^-THC. HGN = horizontal gaze nystagmus

In the present study, subjects were asked to self-assess their level of impairment using a 10-point scale prior to vaporization of ∆^8^-THC and at designated time points post-vaporization. All three subjects reported a zero level of impairment prior to ∆^8^-THC vaporization, and no evidence of HGN was observed in any of these subjects prior to vaporization (see Figs. 2, 3, and 4), despite each of them having measurable levels of Δ^9^-THC in their blood, as would be expected in frequent cannabis users. There was no detection of Δ^8^-THC in blood or exhaled breath prior to vaporization. HGN was evaluated in each subject prior to vaporizing the hemp-derived Δ^8^-THC product and at various time points up to 3 h post-vaporization. The results showed that all three subjects exhibited HGN within the first hour after vaporization. At 60 min post-vaporization, evidence of nystagmus was no longer being exhibited (Figs. 2, 3, and 4). These results correlated with each subject’s self-assessed impairment data, which showed low levels of impairment being reported 60 min after vaporization. At 3 h post-vaporization, all subjects reported a zero level of impairment with no evidence of HGN. These patterns of impairment were similar to those observed in the previous ∆^9^-THC cannabis smoking study (DeGregorio et al. 2021) (Figs. 2, 3, and 4), although there were slight differences in the time points at which self-assessed impairment and HGN were evaluated.

When self-assessed impairment and HGN data for these three subjects were compared to the same data for each subject after they had smoked ∆^9^-THC cannabis in the previous study, the results were consistent with Δ^8^-THC being potentially less psychoactive compared to Δ^9^-THC. Each subject previously smoked a chemovar of cannabis containing 24.6% Δ^9^-THC, which generally produced a higher level of impairment compared to the hemp-derived Δ^8^-THC. In subject 104, the impairment patterns were similar (Fig. 3), while in Subject 105, ∆^9^-THC induced a greater degree of impairment compared to ∆^8^-THC (Fig. 4). In the previous study, subject 103 smoked a low-potency cannabis chemovar (8.5% Δ^9^-THC) followed 60 min later by the higher potency chemovar. Predictably, his impairment levels were lower after smoking the low-potency cannabis compared to the high-potency cannabis, with the impairment induced by the hemp-derived Δ^8^-THC being intermediate between the low- and high-potency cannabis (Fig. 2).

### Cannabinoid kinetic patterns after vaporization of hemp-derived Δ^8^-THC

In both blood and exhaled breath, very short half-lives were observed for Δ^8^-THC within the first hour after vaporization, which is indicative of distribution phase kinetics (see Table 2). Measured over a 20-min period starting immediately after vaporization, the Δ^8^-THC half-lives in the three subjects ranged from 5.2 to 11.2 min in blood. In exhaled breath, the Δ^8^-THC half-lives ranged from 0.8 to 7.8 min based on the consecutive samples collected 15 min after vaporization, which was similar to the ∆^9^-THC half-lives observed in breath 20 min post-smoking in the previous study (see Table 2). No ∆^9^-THC was detected in any of the subject’s samples following ∆^8^-THC vaporization, and no ∆^8^-THC was detected in any subject samples after smoking ∆^9^-THC cannabis in the previous study.

**Table 2:** ∆^8^/∆^9^-THC pharmacokinetic data

	∆^**8**^-THC half-life (min)		∆^**9**^-THC half-life (min)	∆^**8**^-THC breath/blood ratio		
	Time interval post-vaporization/smoking (min)			Time post-vaporization (min)		
**Subject**	**0–20 blood**	**0–15 breath**	**0–20 breath**	**0**	**15**	**180**
103	10.4	0.8	0.4	178.2	46.9	ND
104	11.2	2.8	11.8	12.1	2.3	0.9
105	5.2	7.8	7.3	15.4	20.2	ND

*ND* No detection in blood (limit of detection 0.5 ng/mL), *∆*^*8*^*-THC* ∆^8^-tetrahydrocannabinol, *∆*^*9*^*-THC* ∆^9^-tetrahydrocannabinol

Other cannabinoids detected in exhaled breath within the first hour after vaporization included CBN, THCV, CBC, CBG, and CBD. These compounds were found in the breath of all three subjects except for subject 103, who had no detectable CBG. As observed for Δ^8^-THC, the half-lives for these cannabinoids were short (≤ 5.0 min, where measurable), when determined up to 15 min post-vaporization (see Table 3), which is consistent with distribution phase kinetics. Only CBN showed a somewhat longer half-life of 32.0 min immediately after vaporization in subject 105. Interestingly, CBC and THCV were observed in the breath of all three subjects only within the first hour after vaporization, which is considered the period of peak impairment due to ∆^9^-THC after smoking cannabis, with no detection prior to vaporization. In the previous ∆^9^-THC cannabis smoking study, the same patterns of cannabinoid detection and short half-lives in breath were observed in these subjects in the first hour after smoking (DeGregorio et al. 2021). Trace amounts (< LOQ) of these other cannabinoids were also detected in the subjects’ blood up to 3 h after vaporization.

**Table 3:** Cannabinoid half-lives in exhaled breath after vaporization of hemp-derived ∆^8^-THC

	Half-life (min) 15 min post-vaporization				
Subject	CBN	THCV	CBC	CBG	CBD
103	2.3	2.3	3.7	NC^b^	NC
104	NC	4.9	2.6	NC	2.4^a^
105	32.0^a^	2.7	3.7	NC	1.1^a^

*CBN* Cannabinol, *THCV* Tetrahydrocannabivarin, *CBC* Cannabichromene, *CBG* Cannabigerol, *CBD* Cannabidiol

^a^Determined immediately after vaporization

^b^Not calculable

Another interesting observation concerned the exhaled breath/blood Δ^8^-THC ratios within the first hour after vaporization. This ratio was computed by dividing the ∆^8^-THC peak area ratio to the IS in breath by the corresponding peak area ratio in blood. When determined up to 15 min post-vaporization, the ratios were > 2 in all subjects (see Table 2). In the prior ∆^9^-THC cannabis smoking study, which involved a total of 44 subjects, this phenomenon was observed only within the first hour after smoking for Δ^9^-THC. Beyond the first hour after smoking, these ratios had all fallen below 2.0, with the majority less than 1.0 (DeGregorio et al. 2021). In the present study, only subject 104 had measurable levels of Δ^8^-THC at 180 min post-vaporization, at which time the ratio was 0.9.

## Discussion

Products containing hemp-derived Δ^8^-THC have been increasing in popularity since passage of the Farm Bill in late 2018 legalized U.S. production of industrial hemp, which is legally defined as *Cannabis sativa* plant material containing less than 0.3% dry weight Δ^9^-THC. As isomers, the chemical structures of ∆^8^-THC and ∆^9^-THC are nearly identical; however, comparatively little research has been conducted on ∆^8^-THC and its potential impacts on human health and public safety. Given the rising popularity of hemp-derived Δ^8^-THC products, and the relative lack of knowledge regarding the potential for impairment associated with such products, we wanted to compare patterns of impairment following use of a Δ^8^-THC product to the ∆^9^-THC-induced impairment after smoking cannabis.

In the present study, the patterns of self-assessed impairment and HGN observed in the three subjects were similar to those seen in a previous study in which the same subjects smoked cannabis (DeGregorio et al. 2021), with impairment peaking in the first hour after vaporization and disappearing by 3 h post-vaporization. For cannabis containing ∆^9^-THC, the overall window of impairment is generally agreed to be approximately 3 h after smoking (Huestis et al. 2005; Hartman and Huestis 2013; Couper and Logan n.d.). One notable difference was at 60 min post-vaporization of ∆^8^-THC, where no HGN was observed, while all three subjects were still showing physical evidence of impairment (HGN) 60 min after smoking ∆^9^-THC cannabis. This difference may be due to Δ^8^-THC being less psychoactive compared to Δ^9^-THC (Razdan 1986; Watanabe et al. 1990); however, this does not mean that Δ^8^-THC is any less dangerous with respect to impairment. Unfortunately, to our knowledge, there are no peer-reviewed publications in the medical literature that specifically address the issue of impairment by ∆^8^-THC. The available literature does, however, suggest that ∆^8^-THC and ∆^9^-THC are pharmacodynamically quite similar (Hazekamp et al. 2010; Abrahamov et al. 1995; Kruger and Kruger 2021; Kruger and Kruger 2022), which is consistent with the findings of the present study. Prior to smoking ∆^9^-THC in the previous study, the subjects showed no evidence of impairment despite measurable blood ∆^9^-THC levels, which is consistent with recently published studies showing no significant correlation between impairment and specific blood concentrations of ∆^9^-THC (Brubacher et al. 2019; Hartman et al. 2016; McCartney et al. 2022; Hubbard et al. 2021; Wurz and DeGregorio 2022).

The kinetic patterns observed for ∆^8^-THC and other cannabinoids post-vaporization in the present study were very similar to those observed in these subjects for ∆^9^-THC and other cannabinoids in the previous study (DeGregorio et al. 2021). In both blood and exhaled breath, the half-lives of ∆^8^-THC and ∆^9^-THC were very short within the first hour after vaporization or smoking, which is an indicator of recent use. While ∆^9^-THC half-lives in blood could not be determined 20 min post-smoking in these three subjects in the previous study, the ∆^8^-THC half-lives observed in blood 20 min post-vaporization in the present study were analogous to the ∆^9^-THC half-lives observed in blood 20 min post-smoking in a group of 30 other subjects involved in the previous ∆^9^-THC cannabis smoking study (average 11.3 min, range 4.9–53.2 min; unpublished data). Within the first hour post-vaporization, the ∆^8^-THC breath/blood ratios were > 2 in all subjects, a pattern also observed for ∆^9^-THC in the prior study (DeGregorio et al. 2021), and which may be another indicator of recent use. The pattern of other cannabinoids observed in breath (CBN, THCV, CBC, CBG, and CBD) within the first hour post-vaporization in the present study was likewise consistent with what we observed in these subjects after they had smoked ∆^9^-THC cannabis in the previous study (DeGregorio et al. 2021). Interestingly, CBC and THCV were seen only within the first hour post-vaporization of the ∆^8^-THC product, which suggests that these cannabinoids may be indicators of recent use and, potentially, impairment as we observed in our previous study (DeGregorio et al. 2021), although it should be noted that no impairment window has yet been established for ∆^8^-THC. While the product vaporized by the subjects in the present study was labeled as hemp-derived Δ^8^-THC, it was not surprising to see these other cannabinoids in breath and blood because all of them are commonly found in hemp (Kleinhenz et al. 2020; Abioye et al. 2020).

As designed, the present study has a number of limitations. While the product forms used by the subjects were different in the present study [vaporized hemp-derived Δ^8^-THC concentrate (93%)] compared to the previous ∆^9^-THC study [smoked cannabis flower (24.6% Δ^9^-THC)], cannabis users tend to smoke or vaporize to effect. In other words, regardless of the potency of the product, users will smoke or vaporize until they achieve the desired level of euphoria. In the present study, although the subjects vaporized the ∆^8^-THC product ad libitum with no defined inhalation schedule, they were free to adjust their vaporization patterns to achieve the desired effect. Another limitation of this study was the fact that subjects were not blinded to the product being consumed, which may have affected their self-assessed impairment data. While this effect cannot be ruled out, the HGN observations were consistent with self-assessed impairment, as they were in the previous ∆^9^-THC cannabis smoking study (DeGregorio et al. 2021), suggesting that the subjects were accurately assessing their impairment. Finally, although this study included only three subjects, the results suggest that, based on the observed similarities with Δ^9^-THC from cannabis, impairment resulting from hemp-derived Δ^8^-THC may be a potential safety risk on the roadways and in the workplace. Further study is warranted.

## Conclusions

The patterns of self-assessed impairment and HGN in three case subjects after vaporization of hemp-derived Δ^8^-THC were similar to the ∆^9^-THC-induced impairment patterns observed in the same three subjects after smoking cannabis. The potential for impairment by Δ^8^-THC products derived from hemp, which have been increasing in popularity since the U.S. legalization of industrial hemp in 2018, needs to be considered by employers, law enforcement authorities, and any other agencies or regulatory bodies responsible for setting drug use policy, and Δ^8^-THC needs to be incorporated into standard drug testing panels.

## Data Availability

The datasets generated or analyzed during the current study are available from the corresponding author on reasonable request.

## Abbreviations

AGC: Automatic gain control
CBC: Cannabichromene
CBD: Cannabidiol
CBDA: Cannabidiolic acid
CBG: Cannabigerol
CBGA: Cannabigerolic acid
CBN: Cannabinol
CRM: Certified reference material
DEA: U.S. Drug Enforcement Administration
HESI: Heated electrospray ionization
HGN: Horizontal gaze nystagmus
IS: Internal standard
LC-HRMS: Liquid chromatography high-resolution mass spectrometry
LOQ: Limit of quantification
SFST: Standardized field sobriety testing
Δ^8^-THC: Δ^8^-Tetrahydrocannabinol
Δ^9^-THC: Δ^9^-Tetrahydrocannabinol
Δ^9^-THCV: Δ^9^-Tetrahydrocannabivarin
UHPLC: Ultrahigh-performance liquid chromatography
